# Functional movement screen and asymmetries in female volleyball players across playing positions

**DOI:** 10.1038/s41598-026-35725-w

**Published:** 2026-01-10

**Authors:** Gözde Emir Uysal, Barış Baydemir

**Affiliations:** 1 School of Graduate Studies, Çanakkale Onsekiz Mart University, Çanakkale, Türkiye; 2 Faculty of Sports Sciences, Çanakkale Onsekiz Mart University, Çanakkale, Türkiye

**Keywords:** Volleyball, Female athletes, Functional movement screen, Asymmetry, Injury risk, Anatomy, Health care, Health occupations, Medical research, Risk factors

## Abstract

This study aimed to identify positional differences and asymmetries in functional movement patterns among female volleyball players. A total of 107 professional female athletes from the provinces of Çanakkale and Istanbul participated in the study. Height and body weight measurements were taken to calculate body mass index (BMI), and the Functional Movement Screen (FMS) was administered. Data were analyzed using the SPSS statistical software package. The Wilcoxon Signed Ranks Test was employed for asymmetry analyses, while the Kruskal-Wallis test was used to evaluate differences across playing positions. The findings revealed no statistically significant differences in FMS test scores among players in different positions for deep squat, hurdle step, inline lunge, shoulder mobility, active straight-leg raise, trunk stability push-up, and rotary stability. Regarding injury risk, no significant associations were observed between FMS scores and injury risk for deep squat, inline lunge, active straight-leg raise, and rotary stability; however, statistically significant associations were found for hurdle step, shoulder mobility, and trunk stability push-up. In terms of right-left asymmetries, no significant differences were identified in hurdle step, inline lunge, active straight-leg raise, and rotary stability, while shoulder mobility showed a statistically significant asymmetry between the right and left sides. In conclusion, FMS performance was found to be similar across playing positions among female volleyball players. Additionally, FMS testing appears to be a valuable tool for identifying movement-related indicators and detecting asymmetries in this population. FMS results may be practically applied to support individualized training design and preseason screening in volleyball players.

## Introduction

In today’s world, advancements in technology have led to a noticeable decline in physical activity and motor skills. This trend has also affected the consistency of training among both professional and amateur athletes. As a result, athletes frequently face the risk of injury during training or competitive performance. When such injuries occur, they must undergo treatment and participate in return-to-sport (RTS) rehabilitation to regain their performance levels. The literature contains a considerable number of studies addressing the RTS process following sports injuries^1–4^.

These studies indicate that the primary goal of an injured athlete is to return to sport as quickly as possible^1^. Researchers emphasize the sense of urgency experienced by athletes and define the RTS decision as primarily based on the type and severity of the injury, recovery of symptoms and function, and the athlete’s ability to tolerate the demands of the sport^5,6^.

Volleyball’s complex movements, coupled with movement patterns like jumping and falling that strain the shoulders and lower extremities, place athletes under significant stress. Athletes who are overexposed to training loads are at increased risk of injury or asymmetries, highlighting the importance of FMS in identifying these processes. While injury and RTS studies hold ongoing significance, recent years have seen increasing interest in predicting injury risk before injuries occur. Studies across various sports disciplines aim to identify such risk factors. This issue has also been addressed in football-specific research^7^. The novelty of this study lies in its focus on professional female volleyball players, a group underrepresented in previous FMS research, and its examination of positional differences along with asymmetries. However, a review of the literature shows that there are relatively few studies focused on Functional Movement Screening (FMS)^8,9^. For this reason, the current study aims to investigate positional differences, injury risk, and asymmetries in functional movement patterns among female volleyball players.

The positive effects of regular physical activity on health are well established^10^. However, in performance sports such as volleyball, athletes are required to maintain high levels of physical conditioning^11^. Assessment tools such as FMS have the potential to identify asymmetries and weaknesses in fundamental movement patterns, offering insight into injury risk and ways to mitigate it. The use of such screening methods is crucial for athletes to achieve optimal performance across different playing positions. The novelty value of this research is that it included female volleyball players, an underrepresented group in sports sciences, and analyzed positional differences, asymmetries, and functional movements with the FMS test.

## Methods

### Study design

This descriptive cross-sectional study aimed to investigate positional differences, injury risk, and asymmetries using the Functional Movement Screen (FMS) in professional female volleyball players. As the study did not involve any clinical intervention, registration in an international clinical trials database was not required. The research was conducted in accordance with the principles of the Declaration of Helsinki, and written informed consent was obtained from each participant.

### Participants

The target population comprised professional female volleyball players, with the sample selected from athletes actively competing in Çanakkale and Istanbul, Türkiye. A purposive sampling method was employed for participant selection. Participants were selected based on specific inclusion and exclusion criteria to ensure consistency and reliability in the assessment. The inclusion criteria were as follows: actively competing female volleyball players during the 2023–2024 season in the Turkish Women’s 1st and 2nd Leagues; a minimum of three years of licensed volleyball experience; regular participation in training sessions (at least three times per week); no acute injuries at the time of data collection; and voluntary participation with signed informed consent. Exclusion criteria included having sustained a serious musculoskeletal injury (e.g., ligament rupture or meniscus surgery) within the past six months; presence of chronic orthopedic conditions (e.g., scoliosis, herniated disc); experiencing pain or discomfort preventing completion of the assessment; pregnancy; or refusal to provide informed consent or participate in the test procedures. The study’s sample consisted of clubs from only two provinces (Çanakkale and Istanbul). The findings may be limited in their representativeness. This should be considered a limitation.

### Data collection procedures

A total of 107 professional female volleyball players from clubs in Çanakkale (Çanakkale Belediyespor, Çan Gençlik Kale Spor, and Yeşil Bayramiç Spor) and Istanbul (Vakıfbank Sports Club) participated in the study. Anthropometric measurements—including height, body weight, and body mass index (BMI)—were recorded, and the FMS test was administered.

### Height measurement

Height was measured with participants standing barefoot using a SECA stadiometer (Germany) with a precision of 0.1 cm.

### Body weight measurement

Body weight was measured using a SECA electronic scale (Germany) with an accuracy of 0.05 kg.

### BMI calculation

Body mass index was calculated using the following formula^12^:

$${\text{BMI }} = {\text{ Body Weight }}\left( {{\mathrm{kg}}} \right){\mathrm{/Heigh}}{{\mathrm{t}}^2}{\text{ }}\left( {{{\mathrm{m}}^2}} \right)$$

### Functional movement screening (FMS)

FMS assessments included seven movement tasks: deep squat, hurdle step, in-line lunge, shoulder mobility, active straight-leg raise, trunk stability push-up, and rotary stability. Each movement was scored as follows: 3 points for flawless, pain-free performance; 2 points for compensated but pain-free performance; 1 point for flawed performance; and 0 points for inability to perform or presence of pain. The highest possible composite score was 21, indicating perfect performance across all movements. Total scores of ≤ 14 or less in the FMS test indicate a risk of injury^13,14^.

### Data analysis

Statistical analyses were performed using the SPSS software package. As the sample size exceeded 30, the Kolmogorov–Smirnov test was used to evaluate the normality of the data. Since the data were not normally distributed, non-parametric tests were applied. The Wilcoxon Signed Ranks Test was used to assess asymmetries within participants, and the Kruskal–Wallis test was conducted to compare groups based on playing position. In cases where the Kruskal–Wallis test revealed significant differences, pairwise comparisons were made using the Mann–Whitney U test. The internal consistency of the FMS was evaluated using Cronbach’s alpha coefficient, indicating a reliable measurement tool. The bilateral symmetry index (BSA = |Right − Left| / max (Right, Left) × 100) was calculated using the Wilcoxon Rank Sign test to numerically determine the magnitude of asymmetry. Values ​​above 10% were considered clinically significant asymmetry.

**Table 1:** Cronbach’s alpha reliability coefficient of the Study.

Cronbach’s alpha	*N*
0.728	107

According to Table 1, the Cronbach’s Alpha reliability coefficient for the study was calculated as 0.728 based on a sample of 107 participants. This result indicates an acceptable level of internal consistency for the instrument used in the study, suggesting that the scale items are sufficiently correlated and measure a coherent construct.

## Results

**Table 2:** Demographic characteristics of the participating athletes.

Variables	Group	Mean	Standard deviation	Min.	Max.
Age (years)	Setter	19.80	5.18	15.00	31.00
	Opposite hitter	19.10	3.12	15.00	25.00
	Middle blocker	20.14	5.09	15.00	30.00
	Outside hitter	18.84	3.26	14.00	31.00
	Libero	20.15	3.19	16.00	25.00
Height (cm)	Setter	171.10	5.79	157.00	183.00
	Opposite hitter	173.50	8.53	162.00	188.00
	Middle blocker	182.71	7.96	171.00	196.00
	Outside hitter	175.44	7.14	160.00	188.00
	Libero	166.00	6.62	157.00	176.00
Body weight (kg)	Setter	57.60	7.10	51.00	71.70
	Opposite hitter	62.82	8.53	52.00	77.70
	Middle blocker	63.16	10.83	52.00	85.00
	Outside hitter	61.38	9.26	45.00	78.00
	Libero	56.66	3.93	48.00	66.00
BMI	Setter	19.64	1.80	17.86	24.22
	Opposite hitter	20.79	1.44	19.03	24.16
	Middle blocker	18.75	1.79	16.41	22.60
	Outside hitter	19.90	2.49	16.14	25.56
	Libero	20.57	1.17	19.36	23.53

According to Table 2, when examining the average ages of the players by position, setters and opposite hitters exhibited similar age means, while outside hitters were relatively younger, and middle blockers and liberos were slightly older. In terms of height, middle blockers had the highest average, followed by outside hitters, opposite hitters, setters, and liberos. Regarding body weight, middle blockers were the heaviest group, whereas liberos were the lightest. Setters, opposite hitters, and outside hitters fell in between. According to Body Mass Index (BMI) values, middle blockers had the lowest average, while opposite hitters had the highest. The averages for setters, outside hitters, and liberos were, and respectively. These findings indicate that the physical profiles of volleyball players are closely aligned with the specific physical demands of their playing positions.

**Table 3:** Comparison of FMS scores by playing positions in volleyball Players.

Variables	Group	*N*	Kruskal Wallis	Df	*P*	*n* ^2^
Deep squat	Setter	20	5.635	4	0.228	0.053
	Opposite hitter	20				
	Middle blocker	21				
	Outside hitter	26				
	Libero	20				
Hurdle step	Setter	20	1.457	4	0.834	0.014
	Opposite hitter	20				
	Middle blocker	21				
	Outside hitter	26				
	Libero	20				
Inline lunge	Setter	20	8.885	4	0.064	0.084
	Opposite hitter	20				
	Middle blocker	21				
	Outside hitter	26				
	Libero	20				
Shoulder mobility	Setter	20	1.322	4	0.858	0.012
	Opposite hitter	20				
	Middle blocker	21				
	Outside hitter	26				
	Libero	20				
Active straight-leg raise	Setter	20	4.897	4	0.298	0.046
	Opposite hitter	20				
	Middle blocker	21				
	Outside hitter	26				
	Libero	20				
Trunk stability push-up	Setter	20	7.550	4	0.110	0.071
	Opposite hitter	20				
	Middle blocker	21				
	Outside hitter	26				
	Libero	20				
Rotary stability	Setter	20	4.606	4	0.330	0.043
	Opposite hitter	20				
	Middle blocker	21				
	Outside hitter	26				
	Libero	20				

Effect sizes (η²) are interpreted according to Cohen’s guidelines (~ 0.01 = small, ~ 0.06 = medium, ≥ 0.14 = large).

As shown in Table 3, there were no statistically significant differences in FMS subtest scores across playing positions. None of the movement patterns (deep squat, hurdle step, inline lunge, shoulder mobility, active straight-leg raise, trunk stability push-up, rotary stability) demonstrated meaningful variation between positions.

**Table 4:** Distribution of FMS scores according to injury risks in volleyball players.

Variables	Group	*N*	Rank average	Kruskal Wallis	Df	*P*	Difference	Effect size (*n*^2^/*r*)
Deep squat	0.00	4	49.50	4.538	3	0.209	-	n^2^ = 0.0043
	1.00	20	52.18					
	2.00	37	58.18					
	3.00	46	51.83					
Hurdle step	0.00	1	103.00	13.617	3	**0.003**	**0–1 < 2–3**	n^2^ = 0.128
	1.00	50	52.71					
	2.00	47	52.91					
	3.00	9	61.39					
Inline lunge	1.00	4	62.88	4.026	2	0.134	-	*r* = 0.34
	2.00	86	52.61					
	3.00	17	58.94					
Shoulder mobility	0.00	2	49.50	14.546	3	**0.002**	**0–1 < 2–3**	n^2^ = 0.038
	1.00	2	76.25					
	2.00	95	52.32					
	3.00	8	69.56					
Active straight leg raise	0.00	8	56.19	2.652	3	0.448	-	n^2^ = 0.137
	1.00	48	53.96					
	2.00	32	51.17					
	3.00	19	57.95					
Trunk stability push-up	0.00	8	72.13	10.643	3	**0.014**	**0–1 < 2–3**	*r* = 0.37
	1.00	48	46.02					
	2.00	32	64.61					
	3.00	19	48.66					
Rotary stability	0.00	14	49.50	3.449	3	0.327	-	n^2^ = 0.025
	1.00	61	53.89					
	2.00	26	57.73					
	3.00	6	49.50					

Effect sizes are interpreted according to Cohen’s guidelines (η²: ~0.01 = small, ~ 0.06 = medium, ≥ 0.14 = large; r: ~0.10 = small, ~ 0.30 = medium, ≥ 0.50 = large). Results show correlations between FMS scores and performance categories; direct injury data were not collected.

As shown in Table 4, no significant associations with injury risk were found for the deep squat, row lunge, active straight leg raise, or rotary balance. In contrast, significant associations were observed for the hurdles step (*p* = 0.003), shoulder mobility (*p* = 0.002), and trunk balance push-up (*p* = 0.014). The significant relationships observed in the FMS subtests suggest that lower scores may indicate a potential predisposition to injury, but this relationship cannot be interpreted as causal because direct injury data were not collected in the study.

**Table 5:** Asymmetry scores in FMS test among volleyball players.

Variables		*N*	Rank average	Rank total	z	*p*	Effect size (*r*)
Hurdle step	Negative rankings	22	26.18	576.00	-1.153	0.249	-0.11
	Positive rankings	30	26.73	802.00			
	Binds	55					
Inline lunge	Negative rankings	18	14.00	252.00	-1.732	0.083	-0.17
	Positive rankings	9	14.00	126.00			
	Binds	80					
Shoulder mobility	Negative rankings	27	15.61	421.50	-4.328	**0.000**	-0.42
	Positive rankings	3	14.50	43.50			
	Binds	77					
Active straight leg raise	Negative rankings	18	18.94	341.00	− 0.476	0.634	-0.05
	Positive rankings	17	17.00	289.00			
	Binds	72					
Rotary stability	Negative rankings	14	12.64	177.00	-1.308	0.191	-0.13
	Positive rankings	9	11.00	99.00			
	Binds	84					

Effect sizes (r) are interpreted according to Cohen’s guidelines (~ 0.10 = small, ~ 0.30 = medium, ≥ 0.50 = large).

As shown in Table 5, most FMS subtests revealed no significant asymmetry between the right and left sides. A borderline but non-significant asymmetry was observed in the inline lunge test (*p* = 0.083). The only significant asymmetry was found in the shoulder mobility test, where left-side scores were lower than right-side scores (*p* < 0.001). Bilateral asymmetry index showed that the 10% threshold in shoulder mobility was exceeded, confirming the statistical results.

## Discussion

This study aimed to assess FMS scores, movement asymmetries, and potential injury susceptibility among female volleyball players according to playing positions. The results showed no statistically significant differences in FMS test scores across different positions for deep squat (*p* = 0.228), hurdle step (*p* = 0.834), inline lunge (*p* = 0.064), shoulder mobility (*p* = 0.858), active straight-leg raise (*p* = 0.298), trunk stability push-up (*p* = 0.110), and rotary stability (*p* = 0.330). Regarding injury-related associations, no significant relationships were found for deep squat (*p* = 0.209), inline lunge (*p* = 0.134), active straight-leg raise (*p* = 0.448), or rotary stability (*p* = 0.327). However, significant associations were observed between FMS scores and potential injury susceptibility in the hurdle step (*p* = 0.003), shoulder mobility (*p* = 0.002), and trunk stability push-up (*p* = 0.014) tests, suggesting that these results reflect movement limitations rather than predictive injury risk.

As for right–left asymmetries, no statistically significant differences were detected in the hurdle step (*p* = 0.249), inline lunge (*p* = 0.083), active straight-leg raise (*p* = 0.634), or rotary stability (*p* = 0.191) tests, while a significant asymmetry was found only in shoulder mobility. The asymmetry observed in shoulder mobility may be attributable to repetitive dominant-arm loading, which is consistent with previous studies^15,16^ reporting that repeated use of the dominant arm in volleyball can lead to imbalances in muscle strength and range of motion. Our findings support this and suggest that differences in shoulder mobility are likely due to sport-specific biomechanical loading patterns.

The significant relationship between trunk stability push-up scores and lower performance may be explained by the frequent jumping and landing demands in volleyball, which increase trunk stabilization requirements and neuromuscular control. These sport-specific factors highlight the importance of considering volleyball-related biomechanical loads when interpreting FMS results.

The findings of this study are consistent with previous research reporting limited positional differences in FMS performance among volleyball players^17,18^. However, researchers identified significant deficiencies in core stability in elite volleyball players^19^. These discrepancies may be due to variations in sample size, competition level, and training intensity, suggesting that positional differences could become more pronounced in elite-level cohorts.

The predictive ability of the FMS for injury has been a topic of debate in the literature. Several studies have shown that FMS scores alone are not sufficient to predict injury^9,20^. Our results support this conclusion; while certain associations were found, the FMS cannot be considered a direct predictor of injury risk. Rather, it should be viewed as an assessment tool that reflects movement quality, functional limitations, and asymmetries.

The literature reveals a limited number of studies investigating the relationship between technical skills and FMS performance. However, some studies have addressed the association between technical ability and athletic performance^20,21^ or the predictive value of FMS for injury susceptibility^22,23^. A systematic review examined whether variables such as athlete age, sex, sport type, injury definition, and injury mechanism contribute to inconsistent findings. It was suggested that FMS composite scores and asymmetries may be more predictive of injury susceptibility in older athletes compared to younger ones. Additionally, in athletes from sports such as rugby, ice hockey, and American football, the effect sizes of FMS scores tended to be small^24^.

In one study, FMS scores were used to assess functional capacity and injury susceptibility. The findings indicated that athletes who scored below 17 on the composite FMS had approximately 4.7 times higher odds of experiencing lower extremity injuries during a regular season^25^. Another systematic review evaluated the methodological quality and heterogeneity of studies examining the association between FMS composite scores and subsequent injury risk. It concluded that the predictive value of FMS was insufficient to support its use as a stand-alone injury prediction tool^9^. Similarly, a separate review explored whether FMS scores were associated with future injuries in healthy athletes and found inconclusive results. About half of the included studies reported that lower FMS scores were statistically associated with an increased risk of sports injuries. The heterogeneity in study populations (e.g., athlete type, age, sport exposure) and inconsistency in injury definitions were cited as major barriers to synthesizing the evidence and drawing definitive conclusions^26^.

Researchers have also investigated the predictive power of the FMS, with some findings contradicting previous studies by suggesting that the FMS may not be a useful tool for estimating the risk of musculoskeletal injuries^27^. Another study aimed to determine whether the FMS is a valid predictor of injury among high school athletes and whether a new scoring system could be developed for this population. The results indicated that while the FMS may be helpful in identifying deficiencies in specific movements, it should not be used to predict general injury risk over the course of a season in high school athletes^28^.

Regarding studies on FMS and asymmetries, most have focused on football, with limited research in volleyball. One study examined changes in functional movement patterns over a season among university-level football and volleyball players. The study found no significant changes in overall FMS scores over time or between sports. However, some subtests showed notable differences: performance improved in the Deep Squat and Inline Lunge tests, while scores declined in the Active Straight-Leg Raise and Rotary Stability tests. Additionally, there was a decrease in asymmetry levels and in the number of low-scoring test components by the end of the season^29^.

In another study involving right-hand-dominant female volleyball players in the Croatian Women’s Premier League, researchers investigated whether right–left asymmetries existed in range of motion and movement quality. The results revealed significant differences in the Shoulder Mobility and Active Straight-Leg Raise tests. It was suggested that the superior shoulder mobility in the dominant arm could be attributed to fewer spike and serve repetitions compared to elite-level players^15^.

Further research has shown that shoulder muscle strength asymmetries are commonly observed in volleyball players, largely due to the dominant arm’s frequent use. These asymmetries in the non-dominant arm may contribute to imbalances in strength development and are considered potential risk factors for shoulder injuries^16^.

Some methodological issues should be considered in the findings obtained in this study. Type I error may indicate a lack of adjustment for pairwise comparisons. Another issue is the sample size and the relatively low statistical power of nonparametric tests.

From a practical perspective, these findings indicate that the FMS can be a useful tool in early-season screening of volleyball players. Identifying athletes with low scores in certain FMS movements can allow coaches to develop corrective exercises and targeted training. Furthermore, when integrated with other biomechanical and physiological tests, the FMS can serve as part of a comprehensive model for injury prevention and athlete monitoring.

Overall, this study, focusing exclusively on female volleyball players, integrates positional comparisons with quantitative asymmetry and injury-related indicators. The study provides new findings on functional movement in volleyball and contributes to the literature by offering evidence-based insights for individualized training and injury prevention strategies.

## Data Availability

The datasets generated and/or analyzed during the current study are available from the corresponding author upon reasonable request.
